# The emerging role of IMD 0354 on bone homeostasis by suppressing osteoclastogenesis and bone resorption, but without affecting bone formation

**DOI:** 10.1038/s41419-019-1914-5

**Published:** 2019-09-10

**Authors:** Wenxiang Chen, Ziang Xie, Pan Tang, Yongli Wang, Zhiwei Jie, An Qin, Xuesheng Jiang, Zhijun Hu, Shunwu Fan

**Affiliations:** 1 Department of Orthopaedics, Huzhou Central Hospital, Zhejiang University Huzhou Hospital, Huzhou, Zhejiang China; 2 Department of Orthopaedics, Sir Run Run Shaw Hospital, School of Medicine, Zhejiang University, Hangzhou, Zhejiang China; 3 Key Laboratory of Musculoskeletal System Degeneration and Regeneration Translational Research of Zhejiang Province, Hangzhou, Zhejiang China; 4 Department of Orthopaedics, Shanghai Key Laboratory of Orthopaedic Implant, Shanghai Ninth People’s Hospital, Shanghai Jiaotong University, School of Medicine, Shanghai, China

**Keywords:** Drug delivery, Metabolic disorders

## Abstract

Osteoporosis is caused by an imbalance between bone formation and bone resorption. Receptor activator of nuclear factor-κB ligand (RANKL) promotes the activity and differentiation of osteoclasts via activating the nuclear factor-κB (NF-κB) and mitogen-activated protein kinase (MAPK) signaling pathways. IMD 0354 is a selective molecular inhibitor of inhibitor of NF-κB kinase subunit beta (IKKβ) and effective for treatment of acute and subacute inflammatory diseases through the suppression of NF-κB activation. However, the effect of IMD 0354 on bone homeostasis is unknown. In this study, we demonstrated that IMD 0354 significantly attenuated ovariectomy-induced bone loss and inhibited osteoclastogenesis in mice, whereas bone formation was not affected. Additionally, IMD 0354 dramatically inhibited osteoclast differentiation and function induced by RANKL and macrophage colony-stimulating factor in bone marrow monocytes as verified by tartrate-resistant acid phosphatase (TRAP) staining as well as bone resorption assay in vitro. Subsequently, we found that activation of NF-κB signaling and the ERK/c-Fos axis were blunted during osteoclast formation induced by RANKL. Transcription factors nuclear factor of activated T cells c1 (NFATc1) and c-Fos were suppressed with the decreased expression of osteoclast-related genes by IMD 0354. Our findings suggest that IMD 0354 could be a potential preventive and therapeutic drug for osteoporosis.

## Introduction

Osteoporosis is known as the most common metabolic bone disease and is caused by an imbalance between bone formation and bone resorption^1^, placing a substantial public health burden on the aging population^2,3^. This disorder is characterized by disturbance of the bone microarchitecture leading to diminished bone stability and enhanced risk of fractures^4^.

Osteoclasts are multinuclear giant cells that attach to the bone matrix and play an essential role in bone resorption within the sealing zone. Osteoclast differentiation derived from bone marrow monocytes/macrophages (BMMs) is mediated by receptor activator of nuclear factor-κB ligand (RANKL), a critical cytokine of the tumor necrosis factor (TNF) family, and its receptor RANK^5,6^. TNF receptor-associated factor 6 (TRAF6), which is indispensable for osteoclastogenesis, is activated by RANK–RANKL interaction. Through P62, TRAF6 interacts with protein kinase C (PKC) or TGF-β-activated kinase 1-binding protein 2 (TAB2) and subsequently activates TGF-β-activated kinase 1 (TAK1), resulting in activation of the IκB kinase complex (IKK) to accelerate osteoclastogenesis^7,8^. RANK–RANKL interaction promotes the differentiation of osteoclasts via directly activating several cellular signaling pathways, such as the nuclear factor-κB (NF-κB) cascade and mitogen-activated protein kinase (MAPK) signaling pathway^9^. Additionally, another crucial cytokine, macrophage colony-stimulating factor (M-CSF), is also required for the differentiation of macrophage precursors to mature osteoclasts. Osteoclast function is regulated by several crucial transcription factors, including nuclear factor of activated T cells c1 (NFATc1) and c-Fos. The NF-κB, MAPK, and immune receptor tyrosine-based activation motif (ITAM) signaling pathways, converge to induce the expression of NFATc1, a key regulator that drives osteoclast differentiation, increasing the expression of osteoclast-related marker genes including dendritic cell-specific transmembrane protein (Dc-STAMP) and cathepsin K (CTSK)^10–12^.

Mature osteoblasts are responsible for bone formation through secretion of structural proteins of the bone matrix, collagen type 1 (COL1), and noncollagenous components, such as osteocalcin (OCN). COL1 mineralized by hydroxyapatite crystals plays an essential role in maintaining bone hardness and strength. The activation of the master transcription factor runt-related transcription factor 2 (Runx2) is required for differentiation from preosteoblasts to mature osteoblasts^13,14^. It was reported that the activation of NF-κB decreases bone formation in an estrogen deficiency-induced bone loss animal model^15^. In osteoporosis, proinflammatory cytokines, such as interleukin (IL)-1, IL-6, and TNF, have been shown to be overexpressed by T cells and other cells. The increased cytokines can promote the activation of NF-κB in osteoblasts^16^. This process mainly involves phosphorylation of IKK, causing upregulation of IκBα phosphorylation, followed by elevation of nuclear translocation of the transcription factor P65 to negatively regulate the expression of osteoblast-related genes. Thus, activating NF-κB may lead to acceleration of bone resorption as well as suppression of bone formation^17^.

As mentioned above, targeting IKK upstream can effectively block the NF-κB signaling pathway. Therefore, we screened multiple related compounds and found that *N*-(3,5-bis-trifluoromethyl-phenyl)-5-chloro-2-hydroxy-benzamide (IMD 0354) is a non-ATP binding competitive and selective molecular inhibitor of IKK-β. IMD 0354 has been reported in several studies as an effective agent for the treatment of acute and subacute inflammatory diseases including myocarditis, pulmonary arterial hypertension, and diabetic retinopathy via the suppression of NF-κB activation in various cells, such as pulmonary arterial smooth muscle cells or myocardial cells^18–21^. There are considerable issues in regard to the drugs for osteoporosis treatment currently. For instance, bisphosphonates can markedly inhibit bone resorption and are very efficient at reducing osteoclast numbers and activity, while they also profoundly decrease bone formation^22^. Moreover, lasofoxifene belongs to selective estrogen receptor modulators (SERMS) down-modulating the activity of osteoclasts in a transforming growth factor-β3-dependent manner leading to reduced bone resorption^23^, while it is associated with an approximately two fold increased risk of deep vein thrombosis^24^. As a selective IKK-β inhibitor, IMD 0354 downregulates IκB phosphorylation and blocks NF-κB P65 nuclear translocation. However, the effect of inhibitor IMD 0354 on bone homeostasis is unknown. This drug would work through different mechanisms and might be unique and efficient to prevent and treat osteoporosis. Therefore, we aimed to investigate the effect of IMD 0354 on osteoclast differentiation and function and assess the efficacy of IMD 0354 as a potential therapeutic drug for bone loss in an ovariectomized (OVX) osteoporosis mouse model.

## Results

### IMD 0354 prevents OVX-induced bone loss in vivo

**Fig. 1:**
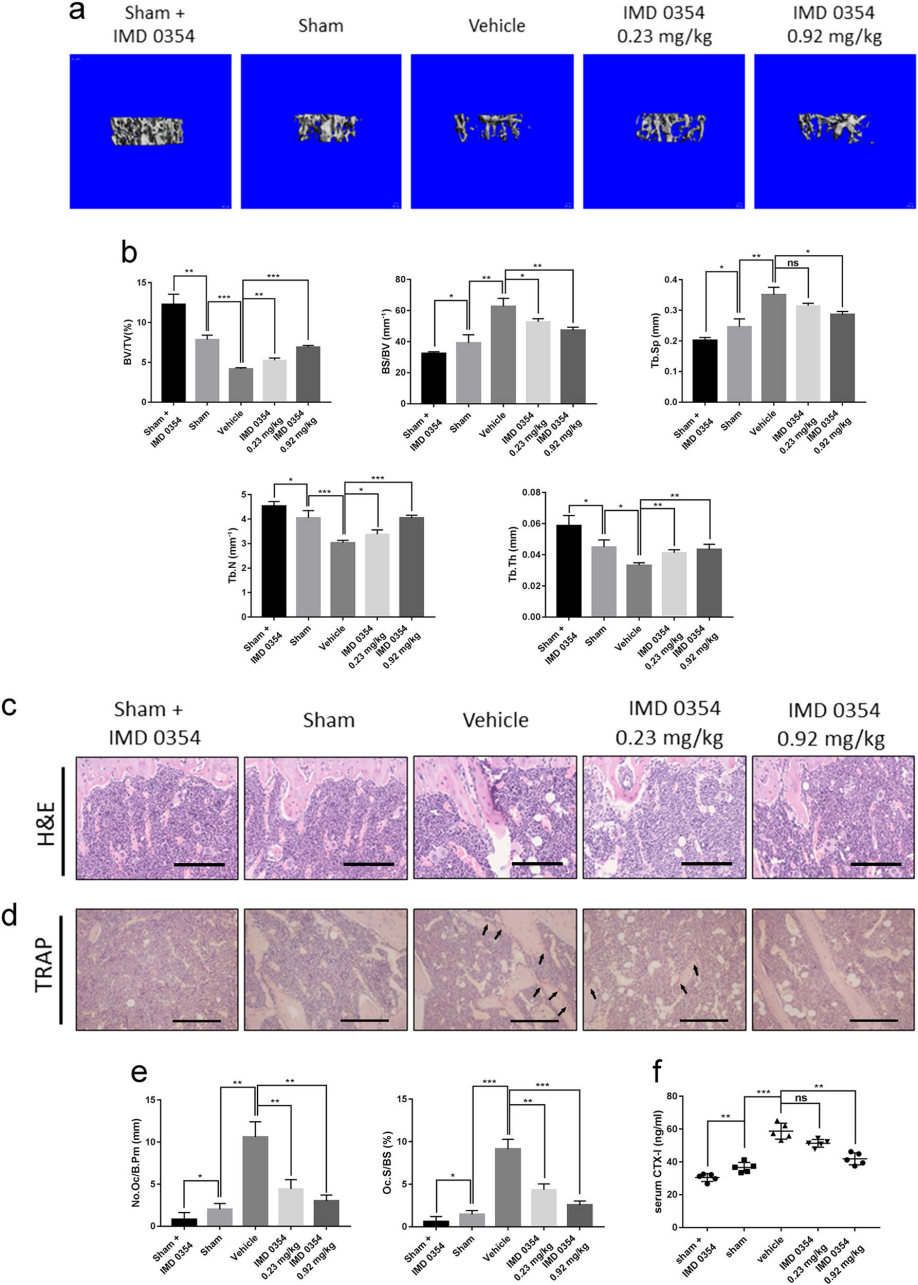
IMD 0354 effectively inhibits OVX-induced bone loss in vivo. **a** The femurs of all mice were scanned with a high-resolution micro-CT. **b** Calculation of the microstructural indices was performed for the micro-CT data. Microstructural indices include bone volume/tissue volume (BV/TV), bone surface/bone volume (BS/BV), trabecular separation (Tb.Sp), trabecular number (Tb.N), and trabecular thickness (Tb.Th). **c** Sections of tibias were stained with H&E. **d**, **e** Sections of tibias were stained with TRAP (black arrows, TRAP-positive cells). The number of osteoclasts per field of tissue (No.Oc/B.Pm) and percentage of osteoclast surface per bone surface (Oc.S/BS%) in sections stained by TRAP. **f** Serum concentrations of CTX-I. Data are presented as mean ± SD (*n* = 5). Scale bars, 50 μm. n.s., no significance, *P < 0.05, **P < 0.01, ***P < 0.005, compared with the vehicle group

We used an OVX-induced mouse osteoporosis model to investigate the effect of IMD 0354 treatment on osteoporosis. The model was successfully established as indicated by the reduced mouse uterus weight and increased body weight (Supplementary Figs. S1a, b). To determine the potential cytotoxic effects of IMD 0354 (Supplementary Fig. S1c) on normal tissues, hematoxylin and eosin (H&E) staining of organs sections collected at the end of the experiment was performed, which indicated no major organ-related toxicity (Supplementary Fig. S1d). All the mice were treated for 4 weeks after OVX operation, and then three-dimensional reconstruction of the images of the femur and spine was performed (Fig. 1a, Supplementary Fig. S2a). Trabecular bone volume per total volume (BV/TV) in the OVX group was shown to be significantly decreased compared with that observed in the sham group due to a decrease of both mean trabecular thickness (Tb.Th), and mean trabecular number (Tb.N), whereas mean trabecular separation (Tb.Sp) and bone surface to bone volume (BS/BV) values were increased in the femur or spine of OVX mice (Fig. 1b, Supplementary Fig. S2b). Following the treatment with IMD 0354, an increase in BV/TV, Tb.Th, and Tb.N values was observed in the OVX-induced mice, compared with those in the vehicle group treated with normal saline. Moreover, BV/TV, Tb.Th, and Tb.N were increased and Tb.Sp and BS/BV were decreased in the sham + IMD 0354 group compared with those in the sham group (Fig. 1b, Supplementary Fig. S2b). Furthermore, histological analysis verified the protective effect of IMD 0354 on OVX-induced bone loss (Fig. 1c, Supplementary Fig. S2c). Apparent bone loss was shown in H&E-stained sections obtained from the OVX groups compared with that in the control group (Fig. 1c, Supplementary Fig. S2c). However, IMD 0354 treatment resulted in an increase in BV/TV in both the low-dose-treated and high-dose-treated group. In addition, TRAP staining showed that the number of multinucleated osteoclasts was significantly increased in the vehicle group compared with that in the sham group, but decreased in the IMD 0354-treated groups compared with that in the vehicle group. The number of multinucleated osteoclasts and the percentage of osteoclast surface per bone surface (Oc.S/BS%) were significantly reduced in the sham + IMD 0354 group compared with those in the sham group (Fig. 1d, e, Supplementary Figs. S2d, e). Osteoclast activity was evaluated by examining the serum levels of bone resorption marker CTX-I. The serum concentrations of CTX-I in the group treated with a high dose of IMD 0354 were remarkably decreased compared with those measured in the vehicle group. The level of CTX-I was reduced in the sham + IMD 0354 in contrast to that in the sham group (Fig. 1f).

**Fig. 2:**
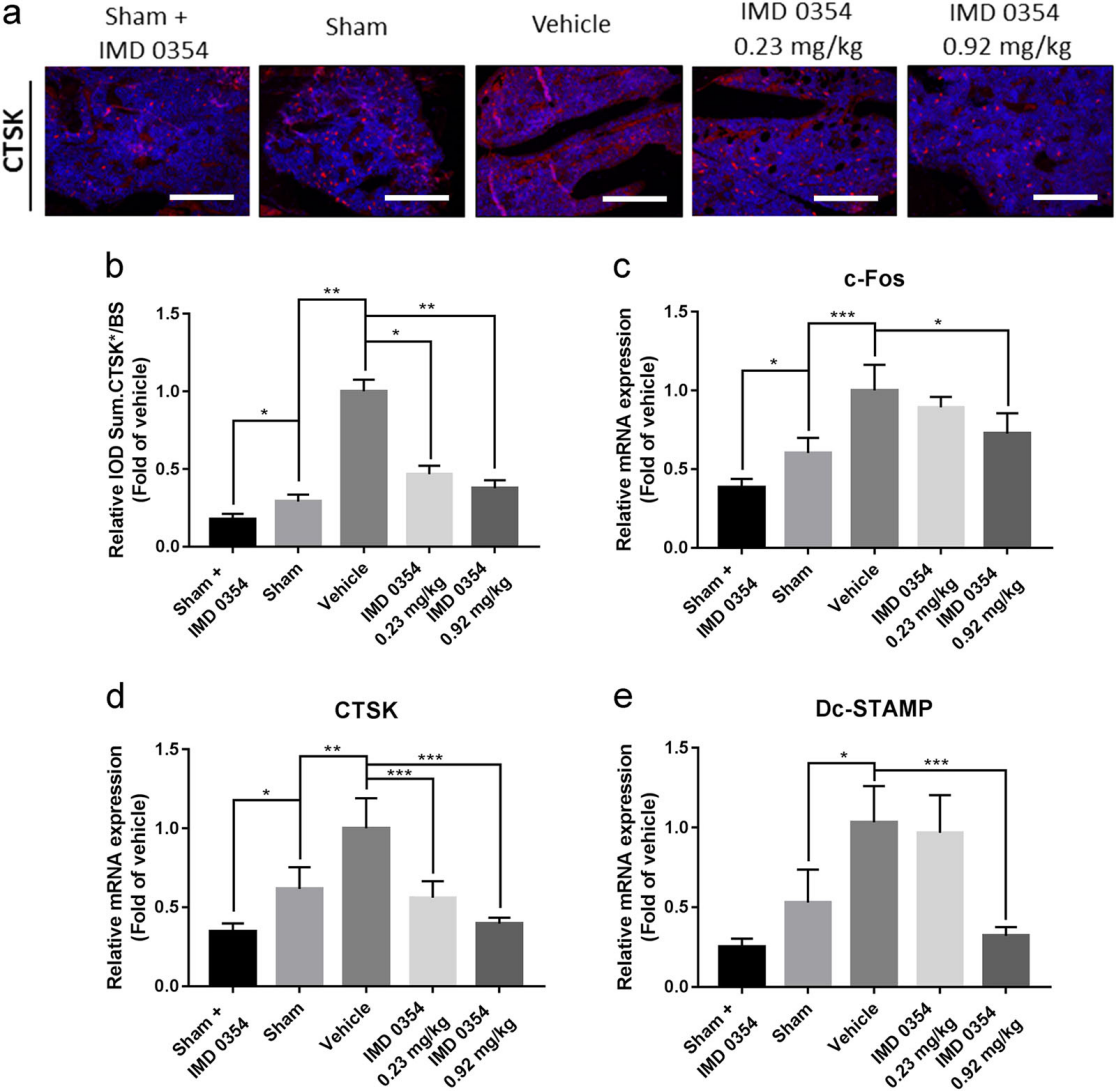
The expression of osteoclast-related genes and protein is suppressed by IMD 0354 in vivo. **a** and **b** Immunofluorescence analysis of CTSK in the tibia sections. Scale bar, 50 μm. **c**–**e** *c-Fos*, *CTSK*, and *Dc-STAMP* mRNA expression in the forelimbs of the treated mice (*n* = 3). Data are presented as mean ± SD. *P < 0.05, **P < 0.01, ***P < 0.005, compared with the vehicle group

Subsequently, we assessed the expression of CTSK in the tibia sections using immunofluorescence analysis (Fig. 2a). It was shown that the expression of CTSK in the low-dose group and the high-dose group was significantly decreased compared with that in the vehicle group. The expression of CTSK in the sham + IMD 0354 group was reduced compared with that in the sham group (Fig. 2b). Then, we examined the expression of osteoclast-related genes including *c-Fos*, *CTSK*, and *Dc-STAMP*. The expression of these genes in the high-dose group was remarkably downregulated in comparison with that in the vehicle group (Fig. 2c–e). Similarly, the expression of *c-Fos* and *CTSK* was significantly downregulated in the sham + IMD 0354 group compared with that in the sham group (Fig. 2c–e). Taken together, these findings indicated that IMD 0354 prevents OVX-induced bone loss in vivo.

### IMD 0354 inhibits bone resorption but does not affect bone formation in vivo

**Fig. 3:**
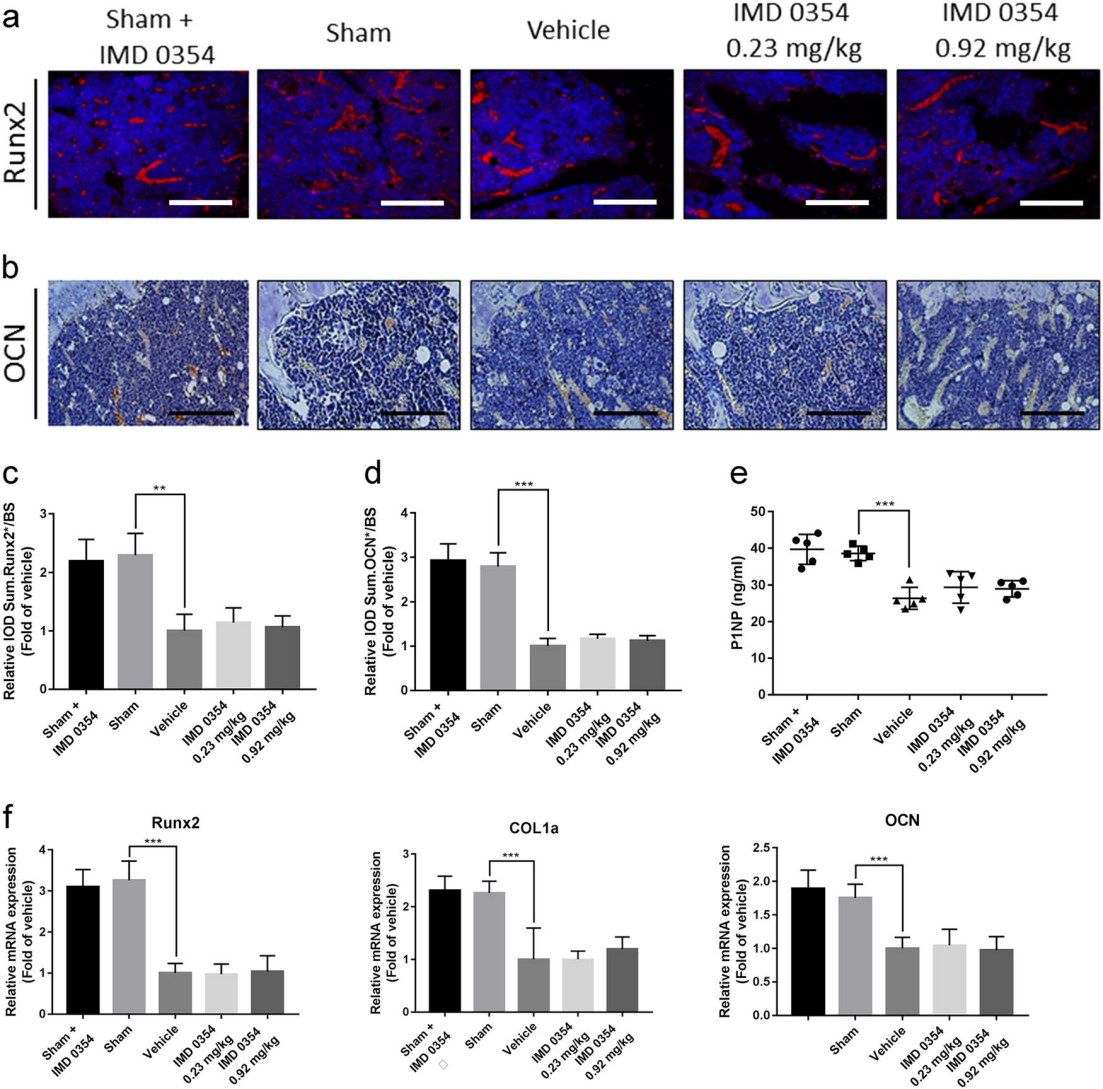
IMD 0354 does not affect bone formation in mice with osteoporosis in vivo. **a** Immunofluorescence analysis of Runx2 in the tibia sections. Scale bar, 50 μm. **b** Immunohistochemical analysis of OCN in the tibia sections. Scale bar, 100 μm. **c** Quantification of immunofluorescence assay of Runx2 (*n* = 5). **d** Quantification of immunohistochemical assay of OCN (*n* = 5). **e** Serum concentrations of P1NP (*n* = 5). **f** *Runx2*, *COL1a*, and *OCN* mRNA expression levels in the forelimbs of the treated mice (*n* = 3). Data are presented as mean ± SD. *P < 0.05, **P < 0.01, ***P < 0.005, compared with the vehicle group

As mentioned above, IMD 0354 plays an effective role in inhibiting bone resorption in vivo. These findings prompted us to examine whether IMD 0354 also exert a role in bone formation. To investigate this possibility, we first examined the expression of Runx2 in the tibia sections using immunofluorescence analysis (Fig. 3a). Then, we assessed the expression of OCN in the tibia sections using immunohistochemical analysis (Fig. 3b). The expression of Runx2 and OCN in the low-dose-treated and high-dose-treated groups was not obviously different from that in the vehicle group. Moreover, the expression of Runx2 and OCN was not changed in the sham + IMD 0354 group after treatment with IMD 0354 (Fig. 3c, d). Consistently, the serum levels of procollagen 1 N-terminal peptide (P1NP) in the low-dose and high-dose groups were not different from those measured in the vehicle group (Fig. 3e). Afterward, we examined the expression of osteoblast-related genes including, *Runx2*, *COL1α*, and *OCN*. The expression of these genes in the low-dose-treated and high-dose-treated groups was not significantly changed in contrast to that in the vehicle group. Likewise, the expression of these genes was not changed in the sham + IMD 0354 group after treatment with IMD 0354 (Fig. 3f). Taken together, these findings suggested that bone formation is not affected by IMD 0354 in vivo.

### IMD 0354 suppresses RANKL-induced osteoclast differentiation and impairs osteoclastic bone resorption in vitro

**Fig. 4:**
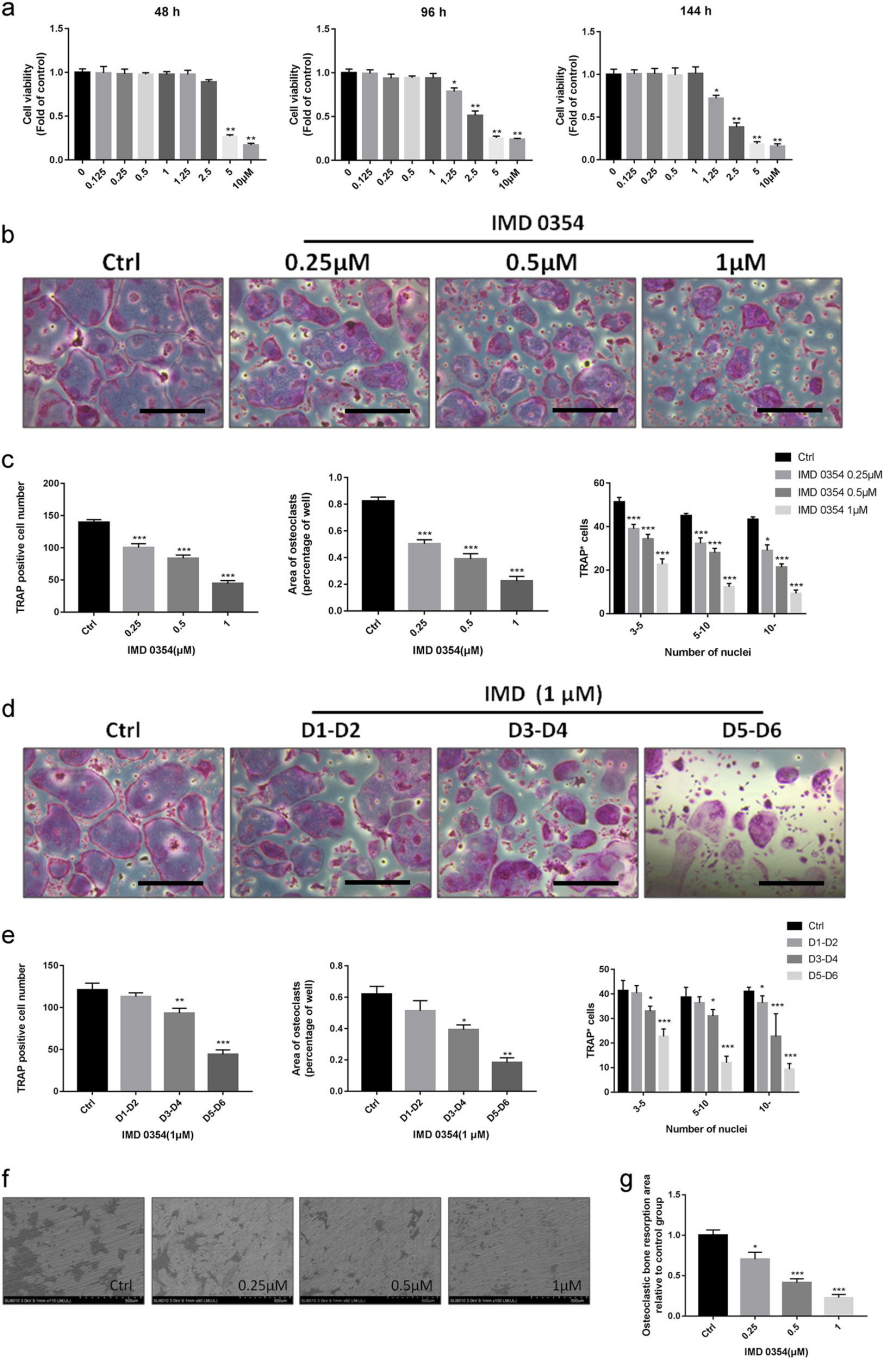
IMD 0354 inhibits the differentiation of osteoclasts in a time- and dose-dependent manner. **a** Effect of IMD 0354 on BMMs viability by CCK-8 assays at 48, 96, and 144 h. **b** BMMs treated with different concentrations of IMD 0354 followed by the stimulation with M-CSF and RANKL for 6 days, cells fixed with 4% paraformaldehyde and stained for TRAP. Scale bar, 100 μm. **c** Quantification of TRAP-positive multinuclear cells including area of osteoclasts, osteoclast number, and osteoclast size. **d** BMMs treated with M-CSF, RANKL, and 1 μM IMD 0354 for the indicated days during osteoclastogenesis. Scale bar, 100 μm. **e** Quantification of TRAP-positive multinuclear cells including area of osteoclasts, osteoclast number, and size. **f** Bone resorption pit images obtained by scanning electron microscope following the treatment with IMD 0354. **g** Resorption pit area measurements using Image J. All experiments were performed at least three times. Data are presented as mean ± SD. *P < 0.05, **P < 0.01, ***P < 0.005, compared with the controls

Since our investigations indicated that IMD 0354 inhibits bone loss in vivo, we postulated that IMD 0354 might have an inhibitory effect on osteoclast formation in vitro. We first performed a cell viability assay to analyze the potential cytotoxic effect of IMD 0354 on BMMs (Fig. 4a). Treatment with 2.5 μM IMD 0354 did not affect the cell viability of BMMs. To evaluate whether IMD 0354 affects RANKL-induced osteoclastogenesis in vitro, we treated BMMs with M-CSF, RANKL, and IMD 0354 at doses of 0.25, 0.5, and 1 μM, respectively. The number, area, and size of osteoclasts were shown to be reduced in a concentration-dependent manner (Fig. 4b, c). In particular, these parameters were dramatically decreased at the late stage of osteoclast differentiation (day 5 to day 6) by IMD 0354 at a concentration of 1 μM. Moreover, a smaller area of osteoclasts, a lower total number of osteoclasts, and a fewer number of osteoclasts with 10 or more nuclei were found compared with those of controls at the middle stage (day 3 to day 4). Intriguingly, treatment with IMD 0354 did not effectively inhibit osteoclast formation in the early stage (day 1 to day 2). Although the size of osteoclasts (nuclei more than 10) was slightly decreased, the number of osteoclasts and the osteoclast area were not significantly decreased compared with those of the control group in the early stage (Fig. 4d, e). Therefore, IMD 0354 inhibited osteoclastogenesis mainly at the late stage of osteoclast differentiation. Additionally, we investigated whether IMD 0354 impaired osteoclastic bone resorption in vitro. IMD 0354 was added to the cell culture medium after BMMs were plated on bovine bone slices in 96-well plates. Treatment with IMD 0354 led to a relative reduction in bone resorption in a concentration-dependent manner (Fig. 4f, g).

**Fig. 5:**
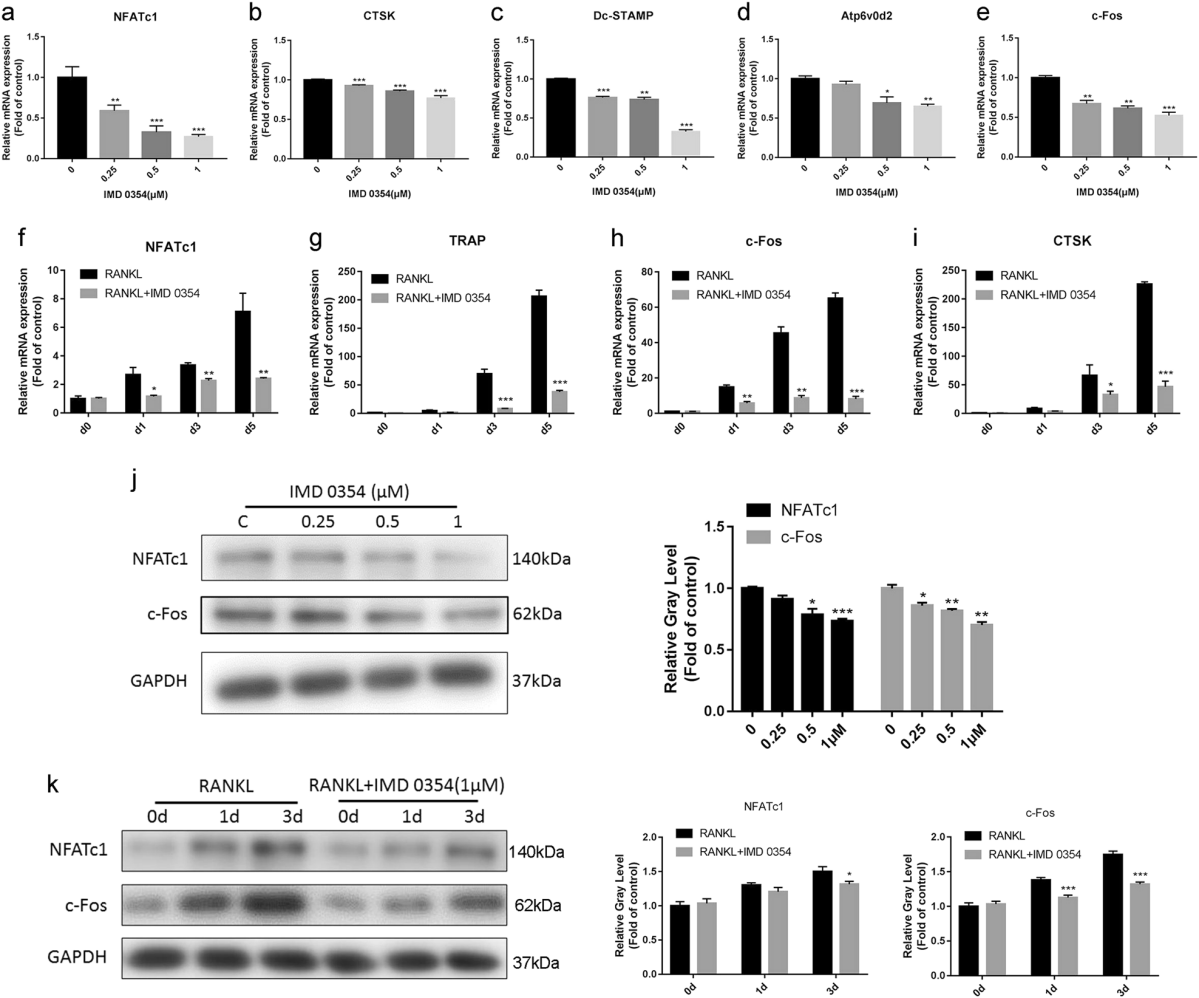
IMD 0354 inhibits RANKL-induced osteoclast-specific mRNA expression in a time- and dose-dependent manner. **a**–**e** qRT-PCR analysis of *NFATc1, CTSK, Dc-STAMP, Atp6v0d2*, and *c-Fos* expression in BMMs treated with the indicated IMD 0354 concentrations for 4 days. Values were normalized to *GAPDH* and negative control expression values (*n* = 3). **f**–**i** qRT-PCR analysis of *NFATc1, TRAP, c-Fos*, and *CTSK* expression in RAW264.7 cells treated with 1 μM IMD 0354 for 0, 1, 3, and 5 days (*n* = 3). **j** Western blot analysis of NFATc1 and c-Fos expression in BMMs treated with various concentrations of IMD 0354 (*n* = 3). **k** Western blot analysis of NFATc1 and c-Fos expression in BMMs treated with 1 μM IMD 0354 for 0, 1, and 3 days (*n* = 3). Values were normalized to GAPDH and negative control expression values. Data are presented as mean ± SD. *P < 0.05, **P < 0.01, ***P < 0.005, compared with the controls

We further investigated the effects of IMD 0354 on the gene expression of osteoclasts. The expression of bone marker genes, including *NFATc1, CTSK, Dc-STAMP*, *Atp6v0d2*, and *c-Fos* was markedly inhibited in a dose-dependent manner (Fig. 5a–e). Furthermore, the expression of bone marker genes, including *NFATc1, TRAP, c-Fos*, and *CT*SK, was inhibited in a time-dependent manner (Fig. 5f–i). Western blot analysis showed that NFATc1 and c-Fos expression in BMMs was decreased in a time- and concentration-dependent manner (Fig. 5j, k), which was consistent with the quantitative reverse transcription polymerase chain reaction (qRT-PCR) analysis results.

**Fig. 6:**
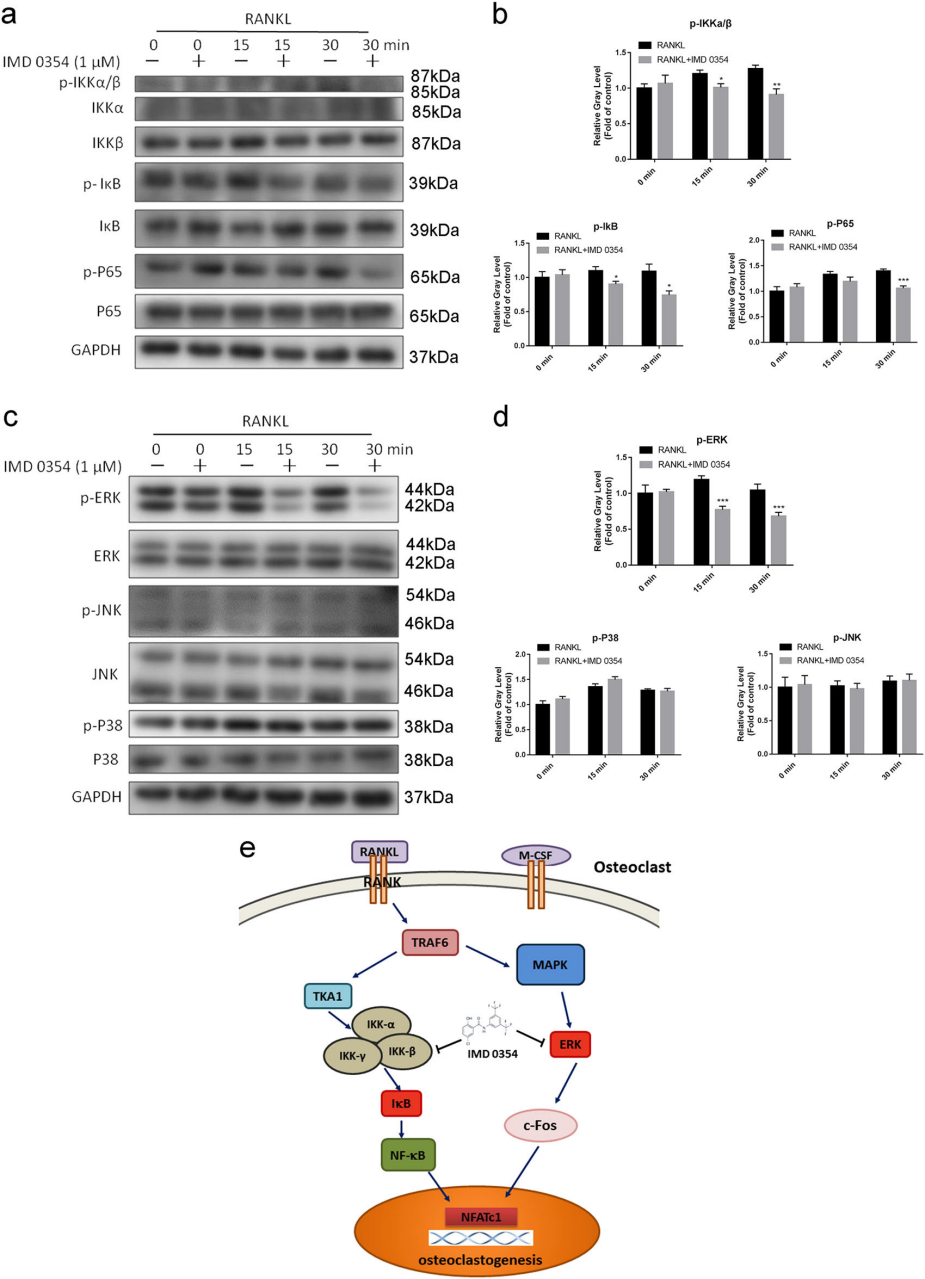
IMD 0354 suppresses osteoclastogenesis by mediating the phosphorylation of IKKβ and IkBα, inhibiting NF-kB nuclear translocation, and blocking the phosphorylation of ERK. **a** BMMs were pretreated with or without 1μM IMD 0354 for 2 h and then treated with 50 ng/mL RANKL for the indicated periods. Cell lysates were analyzed using western blotting. The expression of phosphorylated IKKα/β, IkBα, and P65 and total IKKα, IKKβ, IκBα, and P65 was assessed (*n* = 3). **b** The gray levels of phosphorylated IKKα/β, IκBα, and P65 were quantified and normalized to total IKKα, IKKβ, IκBα, and P65 using Image J (*n* = 3). **c** BMMs were pretreated with or without 1 μM IMD 0354 for 2 h and then treated with 50 ng/ml RANKL for the indicated periods. Cell lysates were analyzed using western blotting. The expression of phosphorylated ERK, P38, and JNK and total ERK, JNK, and P38 was examined (*n* = 3). **d** The gray levels of phosphorylated ERK, JNK, and P38 were quantified and normalized to total ERK, JNK, and P38 using Image J (*n* = 3). **e** Schematic representation of the experiments presented in this image. Values were normalized to GAPDH and negative control expression values. Data are presented as mean ± SD. *P < 0.05, ***P < 0.005, compared with the controls

To unravel the potential mechanisms of IMD 0354 in osteoclast differentiation and bone resorption, we examined the NF-κB signaling pathway. BMMs were treated with RANKL with or without IMD 0354 (1 μM) for 0, 15, and 30 min. We found that phosphorylation of IKKα/β, IκBα, and P65 was repaired compared with that of controls (Fig. 6a, b). Furthermore, we assessed the MAPK signaling pathway including c-Jun N-terminal kinase (JNK), P38, and ERK. The phosphorylation of ERK was drastically blocked after IMD 0354 was added for 15 or 30 min compared with that of controls. However, the phosphorylation of JNK and P38 was not affected (Fig. 6c, d). A schematic representation of the experiments is presented in Fig. 6e. Collectively, these results demonstrated that IMD 0354 plays a negative role in osteoclastogenesis via inhibition of the NF-κB signaling pathway and ERK/c-Fos axis in vitro.

### IMD 0354 does not attenuate osteogenesis in vitro

**Fig. 7:**
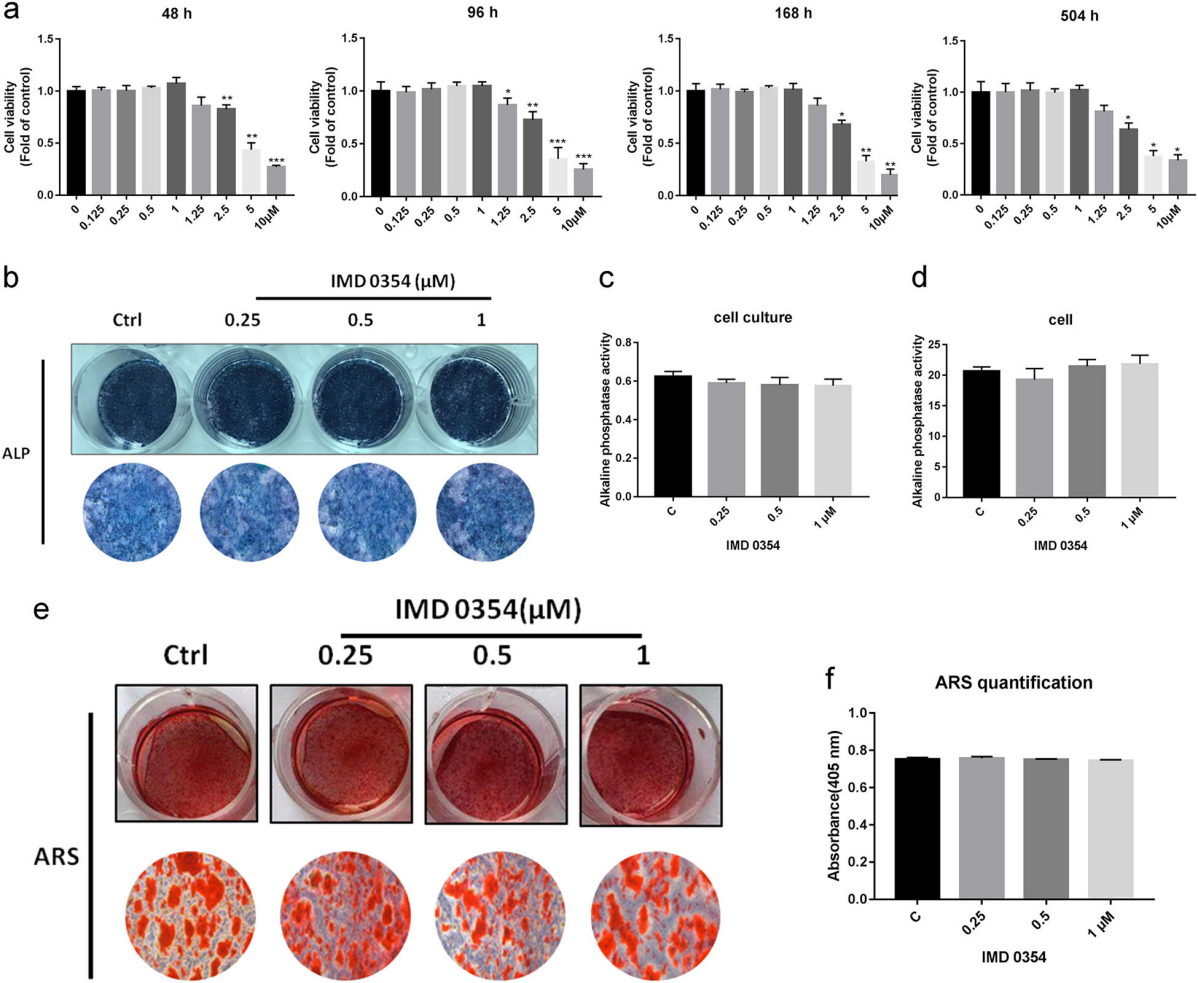
IMD 0354 does not affect osteoblastogenesis. **a** Viability of IMD 0354-treated primary calvarial osteoblasts at 48, 96, 168, and 504 h. **b** ALP expression in primary calvarial osteoblasts cultured in the osteogenic medium after treatment with different concentrations of IMD 0354 for 7 days. **c**, **d** Quantification of ALP in culture medium and cell. **e** Alizarin Red S staining of mineralized extracellular matrix after treatment with different concentrations of IMD 0354 for 21 days. **f** The OD values obtained for mineralized matrix solutions following the treatment with IMD 0354. Data are presented as mean ± SD. All experiments were performed at least three times. *P < 0.05, **P < 0.01, ***P < 0.005, compared with the controls

It was shown that IMD 0354 did not exhibit cytotoxicity toward calvarial osteoblasts at the investigated concentrations (0–1 μM) (Fig. 7a). Then, we assessed the role of IMD 0354 in osteogenesis in the early stage using alkaline phosphatase (ALP) staining (Fig. 7b). Different concentrations of IMD 0354 (0.25, 0.5, and 0.1 mM) did not influence the expression of ALP in the culture medium and calvarial osteoblasts on day 7 (Fig. 7c, d). In addition, Alizarin Red S staining was used to evaluate the effects of IMD 0354 on the deposition of minerals in the extracellular matrix (Fig. 7e). Calcified nodules were not elevated on day 21 following treatment with IMD 0354 (0.25, 0.5, and 0.1 μM), compared with those in the controls (Fig. 7f).

**Fig. 8:**
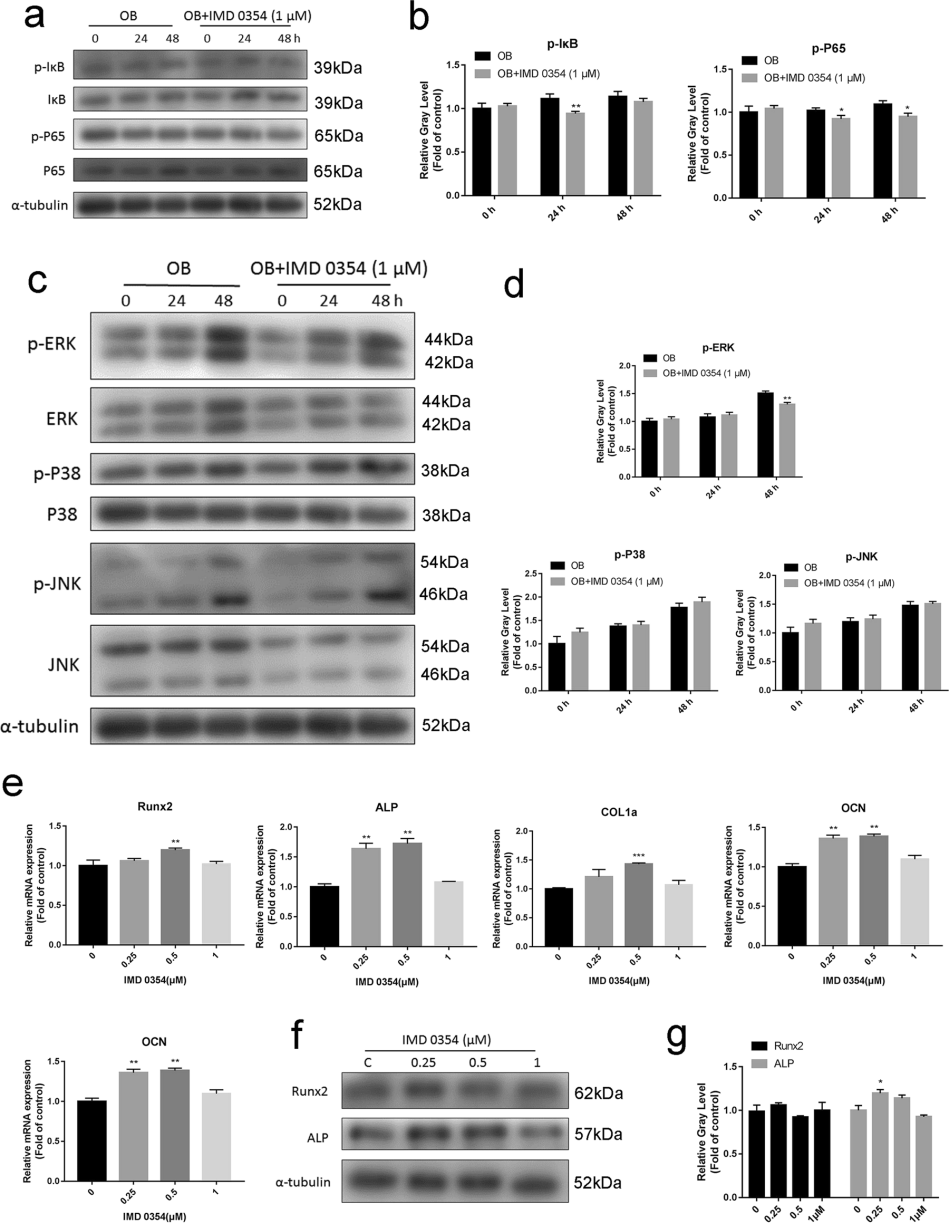
IMD 0354 does not suppress osteoblast-related gene expressions. **a** Primary calvarial osteoblasts were pretreated with or without 1 μM IMD 0354 for 2 h and then treated for the indicated periods. Cell lysates were analyzed using western blotting. The expression of phosphorylated IKKα/β, IκBα, and P65 and total IKKα, IKKβ, IκBα, and P65 was assessed (*n* = 3). **b** The gray levels of phosphorylated IKKα/β, IkBα, and P65 were quantified and normalized to total IKKα, IKKβ, IκBα, and P65 using Image J (*n* = 3). **c** Primary calvarial osteoblasts were pretreated with or without 1 μM IMD 0354 for 2 h and then treated for the indicated periods. Cell lysates were analyzed using western blotting. The expression of phosphorylated ERK, P38, and JNK and total ERK, JNK, and P38 was examined (*n* = 3). **d** The gray levels of phosphorylated ERK, JNK, and P38 were quantified and normalized to total ERK, JNK, and P38 using Image J (*n* = 3). **e** qRT-PCR analysis of *Runx2, ALP, COL1a, OCN*, and *OSX* mRNA expression in calvarial osteoblasts treated with the indicated IMD 0354 concentrations for 4 days (*n* = 3). **f**, **g** Western blot analysis of Runx2 and ALP expression in calvarial osteoblasts treated with the indicated IMD 0354 concentrations for 7 days (*n* = 3). Values were normalized to α-tubulin and negative control expression values. Data are presented as mean ± SD. *P < 0.05, **P < 0.01, ***P < 0.005, compared with the controls

To unveil the underlying mechanisms of IMD 0354 in osteoblast formation, we examined the NF-κB signaling pathway. Calvarial osteoblasts were treated with 1 mM β-glycerophosphate and 5 μM l-ascorbic acid 2-phosphate with or without IMD 0354 (1 μM) for 0, 24, and 48 h. Western blot analyses revealed that the phosphorylation of IKKα/β, IκBα, and P65 was blunted compared with that of the control (Fig. 8a, b). Moreover, we evaluated the MAPK signaling pathway including JNK, P38, and ERK. The phosphorylation of ERK was remarkably inhibited after IMD 0354 treatment for 48 h compared with that of the controls. However, the phosphorylation of JNK and P38 was not significantly changed (Fig. 8c, d).

We further investigated the effects of IMD 0354 on gene expression of calvarial osteoblasts. The results showed that 0.25 μM IMD 0354 enhanced *ALP* and *OCN* mRNA expression, while 0.5 μM IMD 0354 enhanced *Runx2, ALP, COL1a*, and *OCN* mRNA expression slightly on the fourth day (Fig. 8e). However, it was shown that *COL1a, Runx2, ALP*, and *OCN* mRNA expression was not significantly different between the IMD 0354-treated groups and the control group on the 21st day (Supplementary Fig. S3). In addition, IMD 0354 barely increased the protein expression of Runx2 and ALP (Fig. 8f, g). Collectively, these results suggested that osteogenesis is not attenuated by IMD 0354 in vitro.

## Discussion

Osteoporosis therapy and prevention of osteoporotic fractures have been a research focus for many years, yet appropriate drugs with satisfactory curative effects as well as fewer side effects are still in demand. In this study, IMD 0354 was relatively effective in impairing osteoclastogenesis and decreasing bone loss without suppressing osteogenesis and bone formation both in vivo and in vitro. This is the first study to apply the drug IMD 0354 in treatment of OVX-induced osteoporosis.

The cytotoxicity assay suggested that IMD 0354 had no impact on BMMs proliferation. However, osteoclast differentiation was suppressed in a dose-dependent manner in vitro. Consistently, bone resorption assay showed that osteoclast function was negatively regulated by IMD 0354. Furthermore, IMD 0354 noticeably prevented bone loss after 4-week treatment of OVX-induced mice, as shown by H&E staining of the proximal tibia and micro-CT of the distal femur in vivo. As for the potential molecular mechanisms, we verified that IMD 0354 inhibited osteoclast formation by blocking the NF-κB signaling pathway and MAPK cascade in vitro. The NF-κB and MAPK signaling pathways, which are pivotal downstream effectors of RANK–RANKL signaling, regulate osteoclastogenesis^25–29^. In this study, we demonstrated that IMD 0354 suppressed phosphorylation of IKKβ and IκBα, thereby blocking P65 nuclear translocation. NFATc1, a downstream molecule of the NF-κB pathway, is known as a master transcription factor that can regulate osteoclast-specific gene expression to promote osteoclastogenesis and osteoclast function^30–32^. The expression of NFATc1 was reduced in the RANKL-induced osteoclasts treated with various concentrations of IMD 0354 compared with that of the control group. Thus, IMD 0354 inhibited osteoclastogenesis via blunting P65 nuclear translocation leading to downregulated NFATc1 activation. IMD 0354 has been reported to play an inhibitory role in the NF-κB signaling pathway in multiple cell types and effectively treat several diseases. For instance, breast cancer stem cells were targeted by this drug, resulting in decreased breast tumor recurrence^33^. In addition, IMD 0354 inhibited the proliferation of pulmonary arterial smooth muscle cells via blocking the NF-κB signaling pathway, thereby preventing pulmonary arterial hypertension^19^.

Unexpectedly, the ERK signaling pathway was significantly inhibited by IMD 0354, which has not been reported previously. The ERK signaling pathway is involved in many biological processes, including the regulation of inflammation, cell proliferation, and cell differentiation^34,35^. Phosphorylated EAK regulates transcription factor c-Fos, which promotes osteoclast differentiation^36^. Our results also indicated that c-Fos was negatively regulated by IMD 0354, which might be responsible for the repaired osteoclast formation in vitro and attenuated bone loss in vivo. In addition, c-Fos played a critical role in the induction and translocation of NFATc1. In the past several decades, many studies^37,38^ have reported that suppression of the ERK signaling pathway leads to blunted

RANKL-induced osteoclastogenesis. When the activation of NFATc1 and c-Fos was blocked by IMD 0354, the expression of osteoclast-related genes including *CTSK*, *TRAP*, *DC-STAMP*, and *Atp6v0d2* was inhibited both in vitro and in vivo.

In osteoblasts, the NF-κB and ERK signaling pathways were also shown to be inhibited by IMD 0354. Blocking the phosphorylation of P65 directly reduces P65 nuclear translocation, thereby positively regulating multiple cellular signaling pathways, such as activation of Smad1/5/8 phosphorylation or β-catenin signaling, which can promote osteogenesis^39^. In addition, the activation of ERK is critical for osteoblast differentiation and survival^40,41^. However, the phosphorylation of ERK was shown to be suppressed in osteoblasts similarly to osteoclasts. Consequently, our data suggested that osteoblast formation was not influenced by IMD 0354 at a high dose. Based on the theory mentioned above, we speculated that suppressing osteoblast formation by inhibiting the phosphorylation of ERK might neutralize accelerated osteogenesis through repairing the NF-κB signaling pathway. The coupling of inhibition of ERK activation with downregulation of P65 nuclear translocation might be the reason for the lack of effect of IMD 0354 on osteogenesis in vitro and in vivo.

There are some limitations in our study that warrant further investigation in the future. Although we revealed the underlying mechanism of regulation of bone metabolism by IMD 0354, other potential mechanisms remain to be clarified. For instance, our findings raised the question of whether crosstalk between the NF-κB signaling pathway and ERK signaling pathway mediated osteoclast formation in our study. In addition, the detailed mechanisms of the effects of IMD 0354 on osteogenesis remain to be explored.

In conclusion, our study showed that IMD 0354 suppressed osteoclastogenesis and attenuated bone loss in vitro and in vivo. Additionally, we demonstrated that this process is regulated through inhibition of the NF-κB signaling pathway and ERK/c-Fos axis. Hence, our study suggested that IMD 0354 could be a potential preventive and therapeutic drug for osteoporosis.

## Materials and methods

### Reagents and antibodies

IMD 0354 (Fig. 4a) was purchased from Selleck Chemicals (Shanghai, China). Dimethyl sulfoxide (DMSO) was purchased from Sigma-Aldrich (St. Louis, MO, USA). Eagle’s minimal essential medium with alpha modification (α-MEM), fetal bovine serum (FBS), and penicillin/streptomycin were purchased from Gibco-BRL (Carlsbad, CA, USA). Cell Counting Kit-8 (CKK-8) was obtained from Dojindo Molecular Technology (Kumamoto, Japan). Recombinant soluble mouse M-CSF and mouse RANKL were obtained from R&D Systems (Minneapolis, MN, USA). IMD 0354 was dissolved in DMSO and stored at −20 °C. All experiments were performed in the absence of visible light to prevent photosensitivity. IMD 0354 was diluted in cell culture medium so that DMSO comprised <0.1% of the total volume. Specific antibodies against ERK, JNK, P38, phosphorylated p-ERK (Thr202/Tyr204), p-JNK (Thr183/Tyr185), p-P38 (Thr180/Tyr182), IKKα, IKKβ, p-IKKα/β, p-IκBα, IκBα, c-Fos, NFATc1, GAPDH, and β-actin were obtained from Cell Signaling Technology (Beverly, MA, USA). TRAP staining kit and all other reagents were purchased from Sigma-Aldrich, unless otherwise stated.

### Mice

All mice were C57BL/6 background and maintained at the specific pathogen-free animal care facility of Sir Run Run Shaw Hospital, School of Medicine, Zhejiang University. The mice were housed in a room at 24 ± 2 °C, with 50 ± 5% humidity and a 12-h light/dark cycle (lights on from 7:00 a.m. to 7:00 p.m.). All mice were allowed access to water and regular rodent chow ad libitum. All experimental procedures were approved by the Sir Run Run Shaw Hospital Ethics Review Committee for Animal Experimentation.

### BMMs isolation and osteoclast differentiation in vitro

Primary BMMs were isolated from 6-week-old male C57BL/6 mice as described previously^42^. Total bone marrow was flushed out from mouse femurs and tibias. BMMs were resuspended and plated in a 10-cm dish with cell culture medium (α-MEM containing 10% FBS, 1% penicillin/streptomycin, and 25 ng/ml M-CSF). Then, the cells were cultured in an incubator with 5% CO_2_ at 37 °C until they reached 90% confluence. BMMs were seeded in 96-well dishes at a density of 1 × 10^4^ cells per well with 25 ng/ml M-CSF, 50 ng/ml RANKL, and different concentrations of IMD 0354 (0, 0.25, 0.5, and 1 μM). The culture medium was changed every 2 days and cells were cultured for 6 days. Osteoclasts were visualized by TRAP staining according to the manufacturer’s protocol. TRAP-positive cells with more than three nuclei were regarded as osteoclasts.

### Osteoblast differentiation in vitro

Primary calvarial osteoblasts were harvested as described previously^43^. Calvariae were collected from 2- to 4-day-old C57BL/6 mice and digested overnight with collagenase type II in Dulbecco’s modified Eagle’s medium (DMEM) at 37 °C. Then, the cells were collected by centrifugation and seeded into culture dishes in DMEM containing 10% FBS and 1% penicillin/streptomycin. After 4 days, cells were reseeded (2 × 10^4^ cells/cm^2^) in 12-well dishes and cultured in osteogenic medium (1 mM β-glycerophosphate, and 5 μM l-ascorbic acid 2-phosphate) with different concentrations of IMD 0354 (0, 0.25, 0.5, and 1 μM). The medium was changed every 2 days. After 7 days, ALP staining was performed, and the levels of ALP in the cells and medium were determined according to the manufacturer’s instructions (Jiancheng Bio, Nanjing, China). To investigate the effect of IMD 0354 on mineralization, calvarial osteoblasts were seeded at a density of 2 × 10^4^ cells/cm^2^ into 12-well plates. Following treatment with osteogenic medium containing IMD 0354 at concentrations of 0, 0.25, 0.5, and 1 μM for 21 days, cells were gently washed twice with phosphate-buffered saline, fixed in 4% paraformaldehyde for 20 min, and stained with Alizarin Red S solution (Sciencell, San Diego, CA, USA). Untreated cells were considered as a control. Images of extracellular matrix mineralization nodules were obtained using an inverted microscope with a digital camera. Quantification was performed according to the manufacturer’s instructions (Sciencell, San Diego, CA, USA).

### Cytotoxicity assay

To evaluate the effects of IMD 0354 on the proliferation of BMMs and calvarial osteoblasts, CCK-8 assay was used. BMMs were seeded in 96-well plates at a density of 2 × 10^4^ cells per well in triplicate in the presence of 25 ng/ml M-CSF for 24 h. Calvarial osteoblasts were seeded in 96-well plates at a density of 2 × 10^4^ cells per well in triplicate in the presence of osteogenic medium for 24 h as well. Cells were then treated with different concentrations of IMD 0354 (0, 0.125, 0.25, 0.5, 1, 1.25, 2.5, 5, and 10 μM) for 48, 96, or 144 h. Subsequently, 10 μl of CCK-8 buffer was added to each well, and plates were incubated for an additional 2 h. Afterward, the absorbance was measured at 450 nm wavelength (650 nm reference) on an ELX800 absorbance microplate reader (BioTek Instruments, Winooski, VT, USA).

### Bone absorption assay

BMMs were seeded onto bovine bone slices at a density of 1 × 10^4^ cells per well with 50 ng/ml RANKL and 25 ng/ml M-CSF in 96-well dishes. The cells were seeded in triplicate at 0, 0.25, 0.5, and 1 μM IMD 0354 concentrations. After osteoclasts were complete formation, the cells were maintained for additional 3 days. Untreated cells were regarded as a control. Cells were then fixed with 2.5% glutaraldehyde. Subsequently, cells adhered to bone slices were removed by mechanical agitation and sonication. Resorption pits were imaged using a scanning electron microscope (HITACHI, Tokyo, Japan) and the bone resorption area was quantified using Image J software (NIH, Bethesda, MD, USA).

### RNA extraction and qRT-PCR analysis

qRT-PCR was used to assess gene expression levels during osteoclast differentiation. BMMs were seeded in 12-well plates at a density of 1 × 10^5^ cells per well and cultured in the complete α-MEM containing 25 ng/ml M-CSF and 50 ng/ml RANKL. Following the RANKL-induced osteoclastogenesis, BMMs were treated with different doses of IMD 0354 (0, 0.25, 0.5, or 1 μM) for 4 days or with 1 μM IMD 0354 for 0 to 5 days. Total RNA was isolated according to the manufacturer’s protocol. Complementary DNA (cDNA) was synthesized using 1 μg of RNA obtained from each sample, 2 μl of 5× PrimeScript RT Master Mix (Takara Bio, Otsu, Japan), and 4 μl of RNase-free double-distilled water (ddH_2_O) in a total volume of 10 μl. RT-PCR was performed using an ABI Prism 7500 system (Applied Biosystems, Foster City, CA, USA) with SYBR Green QPCR Master Mix (Takara Bio, Otsu, Japan). The total volume (10 μl) of each PCR comprised 5 μl SYBR Green QPCR Master Mix, 2 μl double-distilled water (ddH_2_O), 2 μl cDNA, and 1 μl (10 μM) each of forward and reverse primers. RT-PCR was performed at 95 °C for 10 min (activation), followed by 45 cycles at 95 °C for 10 s, 60 °C for 20 s, and 72 °C for 20 s (amplification), and a final extension at 72 °C for 90 s. Specificity of amplification was verified by performing RT-PCR and analyzing the melting curves. Gene expression assays was normalized to *GAPDH* or *β-actin*. Mouse *GAPDH*, *β-actin*, *CTSK*, *TRAP*, *c-Fos*, *NFATc1, Dc-STAMP*, *Atp6v0d2*, Runt-related transcription factor 2 (*Runx2*), *ALP*, type I collagen *a* (*COL1a*), osteocalcin (*OCN*), and osterix (*OSX*) primer sequences are presented in Supplementary Table S1.

### Western blot analysis

To determine the signaling pathways affected by IMD 0354, BMMs were seeded in six-well plates at a density of 5 × 10^5^ cells per well. The cells were pretreated with 1 μM IMD 0354 for 2 h. Untreated cells were regarded as a control. Afterward, cells were stimulated with 50 ng/ml RANKL for 0, 15, or 30 min. To determine the effects of IMD 0354 on c-Fos and NFATc1 expression, BMMs were treated with 25 ng/ml M-CSF and 50 ng/ml RANKL, and with 0.25, 0.5, or 1 μM IMD 0354 or without it, for 3 or 5 days. To determine the effects of IMD 0354 on Runx2 and ALP expression, primary calvarial osteoblasts were treated with 0.25, 0.5, or 1 μM IMD 0354 or without it, for 7 days. Total protein was extracted from the cultured cells using radioimmunoprecipitation assay lysis buffer (Sigma-Aldrich). Lysates were centrifuged at 12,000 *g* for 10 min, and the supernatants were collected. Proteins were separated by 10% sodium dodecyl sulfate polyacrylamide gel electrophoresis (SDS-PAGE) and transferred to polyvinylidene difluoride (PVDF) membranes (Bio-Rad, Hercules, CA, USA). The membranes were blocked in 5% non-fat dry milk in TBST (50 mM Tris, pH 7.6; 150 mM NaCl; 0.1% Tween 20) at room temperature for 1 h and incubated with the primary antibodies overnight at 4 °C. Protein bands were visualized using LAS-4000 Science Imaging System (Fujifilm, Tokyo, Japan), and the obtained images were analyzed with Image J software.

### OVX-induced osteoporosis model

The animal experiments were performed in accordance with the principles and procedures of the National Institutes of Health (NIH) Guide for the Care and Use of Laboratory Animals and the guidelines for the animal treatment of Sir Run Run Shaw Hospital. An OVX-induced osteoporosis model was established to determine the effects of IMD 0354 on osteoporosis in vivo. Briefly, 25 healthy 8-week-old female C57BL/6J mice were randomly divided into five groups: normal control with a high dose of IMD 0354 (sham + IMD 0354), normal saline control (sham), OVX with normal saline (vehicle), OVX with a low dose of IMD 0354 (IMD 0354-low), and OVX with a high dose of IMD 0354 (IMD 0354-high). After 4 weeks, mice in the IMD 0354-low group were injected intraperitoneally with 0.23 mg/kg IMD 0354 and mice in the sham + IMD 0354 group and IMD 0354-high group were injected intraperitoneally with 0.92 mg/kg IMD 0354 twice per week for another 4 weeks. Mice in the sham and vehicle groups received normal saline. All mice were euthanized at the end of the treatment period of 4 weeks. Body and uterus weights of mice in both groups were determined to confirm the effects of OVX. Vertebral bodies, femurs, and tibias were excised and fixed in 4% paraformaldehyde for histological and micro-CT analyses, respectively. Major organs including heart, liver, spleen, lung, and kidney were obtained and fixed in 4% paraformaldehyde for histological analyses. The forelimbs of mice treated were frozen in liquid nitrogen immediately after dissection for the subsequent RNA extraction and qRT-PCR^44^.

### Micro-CT analyses

The fixed femurs and spine were analyzed using a high-resolution micro-CT (Skyscan 1072, Aartselaar, Belgium) instrument. The scanning protocol was set at an isometric resolution of 9 mm, with X-ray energy settings of 80 kV and 80 mA. After reconstruction, a square region of interest of the distal femur was selected for further qualitative and quantitative analysis. The histomorphometric parameters including BV/TV, Tb.Th, Tb.N, BS/BV, and Tb.Sp were determined for each sample, as reported previously^45^.

### Bone histomorphometry

Histological analysis was performed as reported previously^46^. Histological sections were prepared for TRAP, H&E, immunofluorescence (IF), and immunohistochemistry staining, and the sections were analyzed under a high quality microscope. The number of TRAP-positive multinucleated osteoclasts normalized to the bone area (No.Oc/B.Pm) and surface area of osteoclast to bone surface area (Oc.S/BS) were examined in each sample.

### Enzyme-linked immunosorbent assay

The whole blood was collected by orbital venous of the mice at the end of the treatment period. The mice were fasted before blood drawing. Serum was prepared and stored at −80 °C until use. Enzyme-linked immunosorbent assay was used to measure serum levels of CTX-I (mouse CTX-I ELISA kit; Cusabio, Wuhan, China) and P1NP (mouse P1NP ELISA kit; Cusabio), according to the manufacturer’s instructions.

### Statistical analysis

Statistical analyses were performed using Prism 7 (GraphPad Software, Inc., San Diego, CA, USA). The results are expressed as mean ± SD. Statistical differences were assessed by Student’s *t*-test or one-way ANOVA followed by Tukey’s post hoc analysis. Values of *P* < 0.05 indicated statistical significance.

## Supplementary information

Supplementary Figure S1, S2, S3, Table S1
