# Supplementary material for: The emerging role of IMD 0354 on bone homeostasis by suppressing osteoclastogenesis and bone resorption, but without affecting bone formation
Source: Cell Death Dis. 2019 Sep 10;10(9):654. doi: 10.1038/s41419-019-1914-5 (PMC6737093; doi:10.1038/s41419-019-1914-5)
Supplement: Supplementary file 1 — Supplementary Figure S1, S2, S3, Table S1 [file 41419_2019_1914_MOESM1_ESM.docx]

**
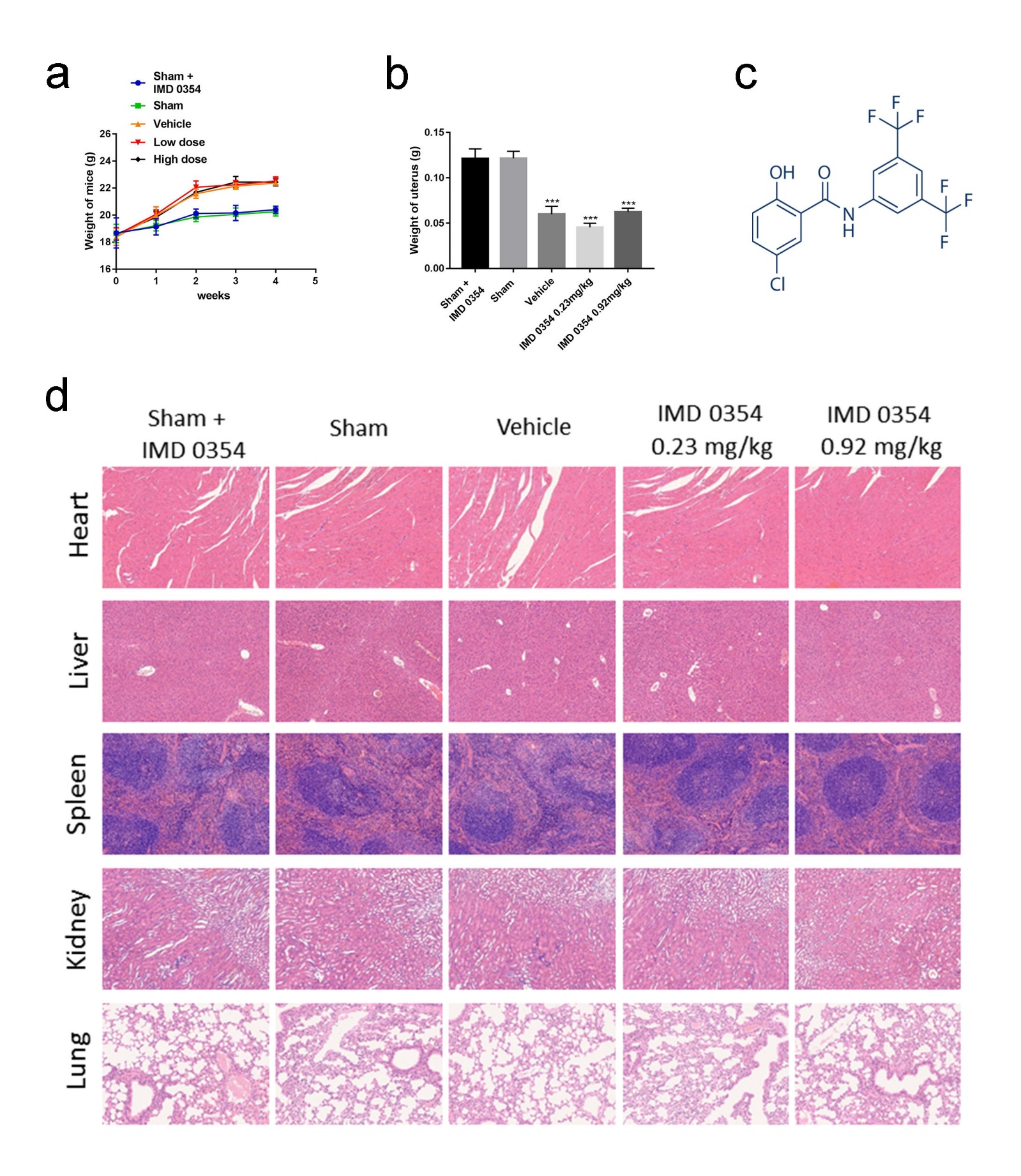
**

**Supplementary Figure S1** (**a**) Body weight of mice after OVX operation at different time. (**b**) Uterus weight of mice after OVX operation at different time. Data is presented as mean ± SD (*n* = 5). **P* < 0.05, ***P* < 0.01, ****P* < 0.005, in contrast to the sham group. (**c**) Chemical structure of IMD 0354. (**d**) No major organ-related toxicities was observed. The histology was evaluated with H&E staining (*n* = 5). Scale bars, 100 μm.

**
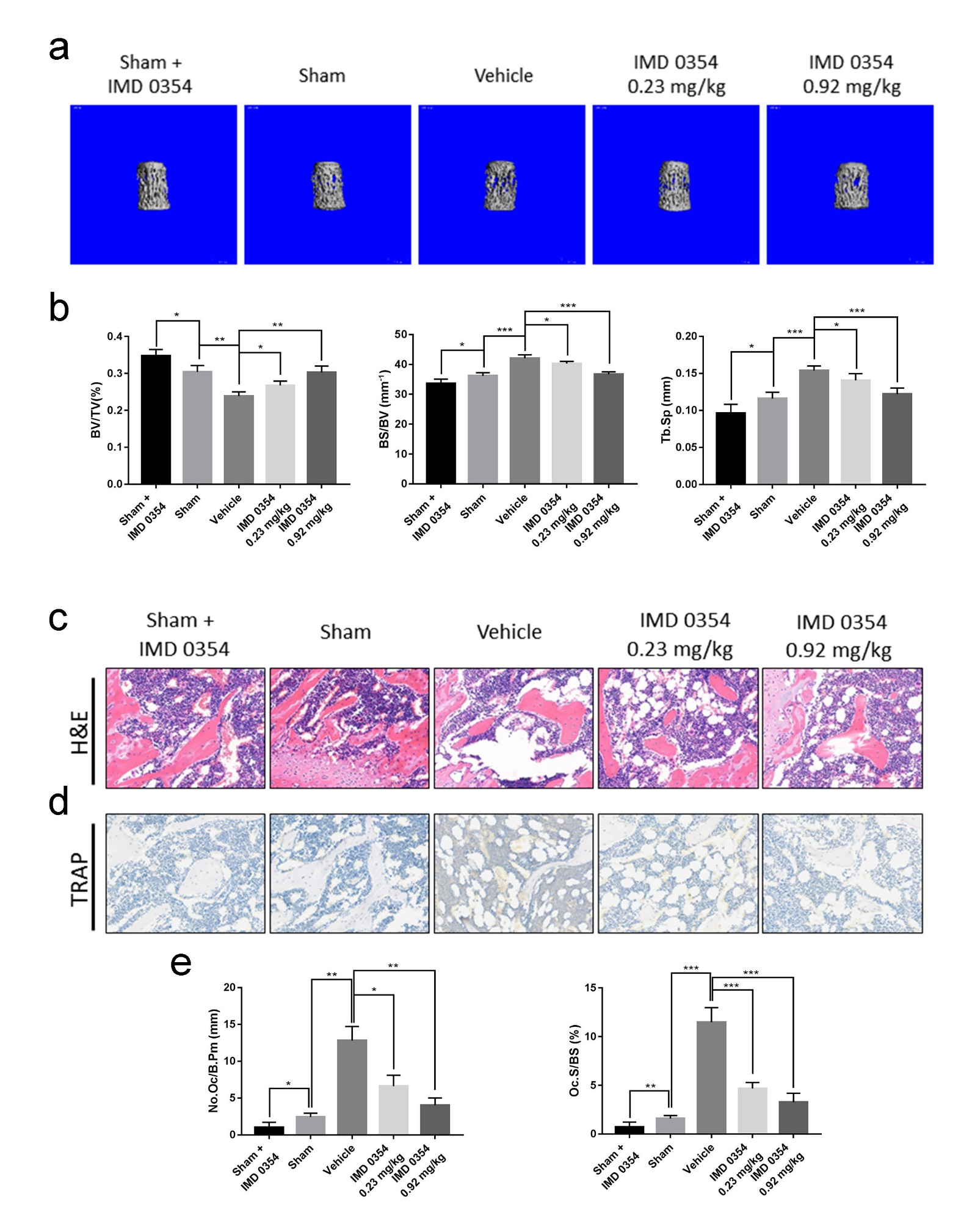
**

**Supplementary Figure S2** (**a**) The lumbar of all mice was scanned with a high-resolution micro-CT. (**b**) Calculation of the microstructural indices was performed for the micro-CT data. Microstructural indices include bone volume/tissue volume (BV/TV), bone surface/bone volume (BS/BV), trabecular separation (Tb.Sp). (**c**) Sections of femurs were stained with H&E. (**d** and **e**) Sections of femurs were stained with TRAP. The number of osteoclasts per field of tissue (No.Oc/B.Pm) and Oc.S/BS% in sections stained by TRAP. Data is presented as mean ± SD (*n* = 5). Scale bars, 50 μm, **P* < 0.05, ***P* < 0.01, ****P* < 0.005, in contrast to the vehicle group.

**
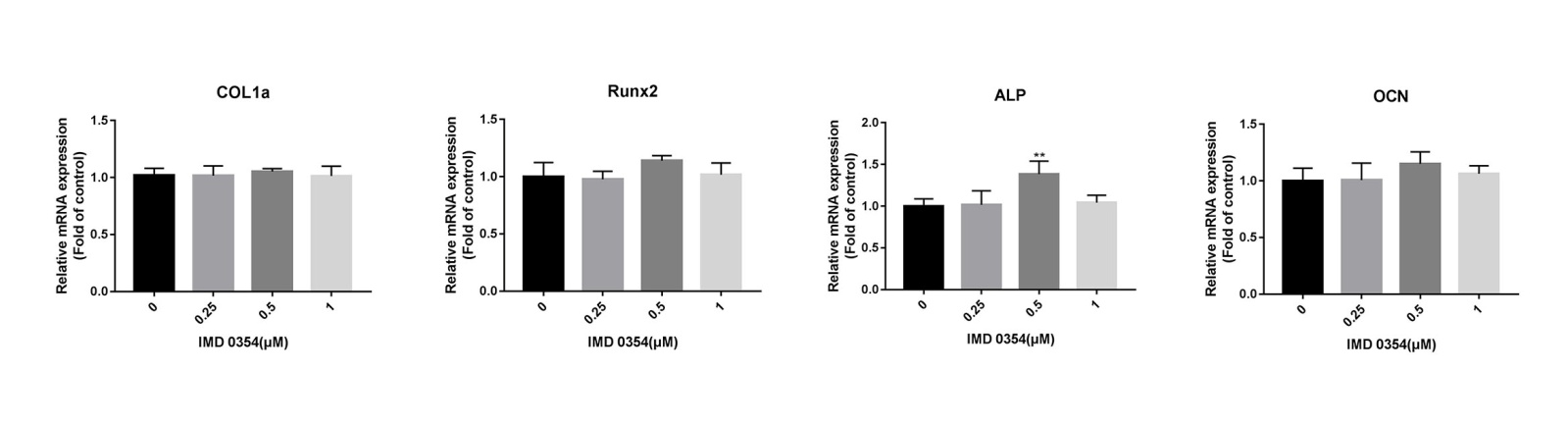
**

**Supplementary Figure S3** qRT-PCR analysis of *COL1a, Runx2, ALP,* and *OCN* mRNA expression in calvarial osteoblasts treated with the indicated IMD 0354 concentrations for 21 days, *n* = 3, compared with the controls. **P* < 0.05, ***P* < 0.01, ****P* < 0.005.

**Supplementary Table S1. Primers used in the real-time PCR**

| **Genes** | **Upstream (5′-3′)** | **Downstream (5′-3′)** |
| --- | --- | --- |
| ***GAPDH*** | ACCCAGAAGACTGTGGATGG | CACATTGGGGGTAGGAACAC |
| ***CTSK*** | CTTCCAATACGTGCAGCAGA | TCTTCAGGGCTTTCTCGTTC |
| ***TRAP*** | CTGGAGTGCACGATGCCAGCGACA | TCCGTGCTCGGCGATGGACCAGA |
| ***c-Fos*** | CCAGTCAAGAGCATCAGCAA | AAGTAGTGCAGCCCGGAGTA |
| ***NFATc1*** | CCGTTGCTTCCAAAAATAACA | TGTGGGATGTGAACTCGGAA |
| ***Dc-STAMP*** | AAAACCCTTGGGCTGTTCTT | AATCATGGACGACTCCTTGG |
| ***Atp6v0d2*** | GACCCTGTGGCACTTTTTGTATTC | GCTTGCATTTGGGGAATCTATC |
| ***ALP*** | CCAACTCTTTTGTGCCAGAGA | GGCTACATTGGTGTTGAGCTTTT |
| ***COL1a*** | CCCAGAGTGGAACAGCGATT | ATGAGTTCTTCGCTGGGGTG |
| ***OCN*** | GAGGGCAATAAGGTAGTGA ACAGA | AAGCCATACTGGTTTGATAGCTCG |
| ***Runx2*** | TTCTCCAACCCACGAATGCAC | CAGGTACGTGTGGTAGTGAGT |
| ***OSX*** | ATGGCGTCCTCTCTGCTTG | TGAAAGGTCAGCGTATGGCTT |
